# The Implicit Ecosystem of Outdoor Therapies: A Grounded Theory Exploratory Study of International Practitioners’ Guiding Frameworks and the Proposition of a Practice Theory

**DOI:** 10.3390/ijerph23030394

**Published:** 2026-03-20

**Authors:** Carina R. Fernee, Markus Mattsson, Pekka Lyytinen, Nevin J. Harper

**Affiliations:** 1 Department of Child and Adolescent Mental Health, Sørlandet Hospital, Egsveien 100, 4615 Kristiansand, Norway; markus.tapani.mattsson@sshf.no; 2 Department of Sport Science and Physical Education, University of Agder, Universitetsveien 25, 4630 Kristiansand, Norway; 3 Muutosloki Turku Ay, Yliopistonkatu 26b, 20100 Turku, Finland; pekka.lyytinen@muutosloki.fi; 4 School of Exercise Science, Physical and Health Education, University of Victoria, P.O. Box 1700 STN CSC, Victoria, BC V8W 2Y2, Canada; njharper@uvic.ca

**Keywords:** outdoor therapy, nature-based, implicit theory, grounded theory, ecosystem

## Abstract

**Highlights:**

**Public health relevance—How does this work relate to a public health issue?**

- Mental and physical health concerns increasingly exceed healthcare system capacity, necessitating identification of accessible, low-cost health promotion and treatment strategies that can improve population health outcomes.
- Outdoor therapies offer biopsychosocial approaches to address stress-related and mental health concerns with broad applicability across populations and settings.

**Public health significance—Why is this work of significance to public health?**

- This international study elicits implicit therapeutic frameworks from 68 practitioners across 18 nations, proposing a practice theory comprising eight interrelated components that describe the therapeutic mechanisms of outdoor therapies.
- The proposed practice theory can be applied to diverse populations along the continuum of care from health promotion to specialized treatment, and across varied nature environments and cultural contexts.

**Public health implications—What are the key implications or messages for practitioners, policy makers and/or researchers in public health?**

- Outdoor therapies provide dynamic and multi-dimensional therapeutic approaches adaptable to physical, mental, emotional, social, behavioral, and cultural needs.
- Outdoor therapies can be applied as self-care, health promotion, health care and rehabilitation, and as such offer versatile approaches that can be implemented along the continuum of care from preventive strategies across to specialized treatment.

**Abstract:**

Human health and well-being are dependent on natural environments, which is the core foundation of the growing discipline of outdoor therapies. However, as with psychotherapy research in general, the field of outdoor therapies lacks descriptive process-oriented theoretical frameworks that precisely reflect this multi-faceted practice. Therapeutic work, whether this takes place indoors or outdoors, comprises numerous implicit relational and environmental dimensions. Implicit aspects are largely sensed, embodied and intuitive, and therefore hard to pin down and describe accurately. In this exploratory study, a survey mapped implicit guiding frameworks amongst outdoor therapy practitioners (*n* = 68) representing 18 nations. A constructivist grounded theory analysis resulted in the proposition of a practice theory, called *the implicit ecosystem of outdoor therapies*, made up of eight interrelated components: (1) joint engagement and co-creating agendas; (2) a foundation of safety and trust; (3) being in parallel and not fix; (4) awareness and attunement here-now; (5) the dynamic of outer and inner landscapes; (6) a constantly moving and meaning-making endeavor; (7) creativity, play, and whole-body activation; and (8) working through natural barriers and rewriting narratives. This grounded theory offers a preliminary blueprint of a practice-guiding framework developed from within the outdoor therapy discipline intended to advance theory, training, and research.

## 1. Introduction

The health benefits and therapeutic potential of nature contact are well established [1,2,3] and recent years have seen an exponential growth in outdoor therapies and nature-based health initiatives [4]. A plethora of terms depict the range of intentional therapeutic practices taking place outdoors, including adventure therapy [5,6,7], wilderness therapy [8,9], nature-based therapy [10,11,12,13], ecotherapy [14,15,16,17], and forest therapy [18,19,20] among other iterations. Outdoor therapies, used here as an inclusive umbrella term, have been defined as comprising three elements: (a) a place-based practice generally located outdoors in intentionally chosen nature environments, (b) an embodied and actively engaging, experiential practice, that happens (c) in kinship with nature [21]. Outdoor therapies can be situated on a time and place continuum from relatively short or single-day sessions in nearby nature areas, to multi-day expeditions in more remote terrain [21]. Around the globe, a diverse range of outdoor therapy practices are facilitated to meet physical, mental, emotional, social, behavioral, and cultural needs, and are suggested to comprise five domains, including: (a) biological, (b) psychological, (c) socio-cultural, (d) ecological, and (e) ethical therapeutic practices [22]. Together these elements, applied in nature environments, offer a dynamic and multi-dimensional therapeutic milieu. Ethics should be placed at its core, constantly guiding decision-making and differentiating safe from harmful practices [23,24], which requires additional considerations when therapy is recontextualized from indoor to outdoor environments [25].

Depending on the intentions, content, context, and participant(s) of a given practice, the provision of outdoor therapies requires an appropriate integration of necessary competencies across health, psychology, outdoor, and activity-specific dimensions to ensure the continuous safety and well-being for each individual participant [4], and to minimize harm to the environment [24]. The practice of outdoor therapies relies on clinical experience and a steadily growing evidence base, and to date, primarily, on research and theoretical frameworks developed within related disciplines spanning psychology, physiology, biology, ecology, philosophy, outdoor and experiential education, in addition to cultural and traditional approaches [26]. Whereas most providers of outdoor therapies express some variant of theoretical foundations and guiding frameworks, mechanisms that bring about change are still incompletely understood [24]. One reason for this is that therapeutic change often occurs in the so-called implicit or tacit domain of practice [27].

In the context of psychotherapy, the implicit domain refers to therapists’ internal working models and the manifestations of these, which are not identical to explicit theoretical frameworks. Cognitive behavior therapy [28], as an example of the latter, may constitute a given practitioner’s therapeutic approach and techniques that guide a therapist step-by-step through a session; directing the way they phrase their questions and respond to clients’ expressions or actions. The more implicit dimensions are the practitioners’ tacit relational “knowing” of how to “be with an other” [29] (p. 1051), where therapeutic change is suggested to result from interactional, intersubjective, and non-linear processes between facilitators and participants. In the context of outdoor therapies, the facilitator–participant dyad is expanded to an interactional triad between facilitator, participant, and nature, where multi-directional microprocesses occur simultaneously. This dynamic therapeutic milieu can be compared to an ecosystem, where each contributing factor is hard to single out and pin down, and as such ought to be understood and described through a systemic lens.

Research endeavors into tacit therapeutic “landscape” is not an easy undertaking for several reasons [30]. First, implicit frameworks exist in a dialogue and on a continuum with more explicit forms of cognition [31]. Second, the intuitive and improvisational qualities of the implicit terrain may render it inaccessible, which brings us to the choice of research methods that lend itself to implicit investigations. Such in-depth enquiries can take place at local levels through context-sensitive case studies [29,32]. However, considering that the field of outdoor therapies represents vast diversity in practice, a first step could be an initial mapping of practitioners’ guiding frameworks as a broader starting point. Theory-generating approaches, such as constructivist grounded theory [33,34], are particularly helpful when attempting to develop new conceptual frameworks from within a discipline and to “move beyond description through constructing new concepts that explicate what is happening” [35] (p. 307). In the present study, an international survey invited practitioners to describe their implicit frameworks, where the overall purpose was to construct a practice theory for outdoor therapies.

## 2. Materials and Methods

### 2.1. Study Setting and Data Collection

This exploratory study was administered through the University of Victoria, Canada, by the principal investigator (N.J.H.) supported by an international team of researcher-practitioners from Norway (C.R.F.) and Finland (M.M., P.L.). The online survey was built and distributed from the University of Victoria’s institutional account on Survey Monkey Inc., Ottawa, ON, Canada and was widely distributed via national and international social media and professional network platforms, such as LinkedIn, Facebook, and formerly Twitter, inviting outdoor therapy practitioners to participate. This snowball sampling took place over a three-month period from March to May 2023. The online survey first collected demographic information including age, gender, nationality, primary and secondary field of practice, years of practice, primary and secondary target populations, and a description of how respondents became an outdoor therapy practitioner. Next, followed the main open-ended question: “What implicit theories guide outdoor therapies?” which invited respondents to share their practice frameworks as free text and as such providing the elicited data for this study. Implicit theories were described as: “Those that are not explicit. It is those practices that promote/lead to/instigate/or create circumstances for client change”. To strike a balance between ensuring that the respondents understood the term “implicit theory” and to avoid leading their responses, a single illustrative example included: “How walking pace, stride length, and terrain choices can increase/decrease attunement for better client–counsellor connection”. To be included in the study, survey respondents had to: (a) self-identify as outdoor therapy practitioners, (b) provide their informed consent, and (c) complete the survey.

### 2.2. Participants

A total of 93 survey responses were submitted. Average completion time was 12 min and 54 s. Survey responses that were not complete, i.e., did not respond to the open-ended question (*n* = 18), along with responses that merely provided keywords (*n* = 7), were excluded. The final sample included 68 full-text responses representing 18 nations. Respondents originated from Canada (*n* = 19), New Zealand/Aotearoa (*n* = 14), Australia (*n* = 5), Finland (*n* = 4), Spain *(n* = 4), and the United Kingdom *(n* = 4). Three or less respondents originated from Austria, Germany, Iceland, Ireland, Israel, Latvia, Lithuania, Norway, Poland, Sweden, Taiwan, and the USA. Thirty-eight respondents identified as women, 29 as men, and one respondent as non-binary/non-conforming. Respondent ages ranged from 25 to 65 years, with a mean age of 41,4 and a median age of 40 years. The sample represented professionals from different fields of practice and various educational backgrounds. Several respondents had attained master’s level education (*n* = 32), slightly fewer had a bachelor’s degree (*n* = 18), and some had a doctoral degree (*n* = 6). Twelve respondents had a non-academic degree. The most common field of study was psychology (*n* = 21), closely followed by counseling (*n* = 16). There were roughly as many licensed therapists (*n* = 13) as specialists in education (*n* = 11) and social work (*n* = 11). Several respondents (*n* = 27) had additional outdoor-related degrees or certifications, such as climbing or kayaking instructor, or qualifications related to wilderness first aid. One respondent reported having no relevant background, while many (*n* = 28) had either a primary education or an additional degree in another field such as medicine, law, or occupational therapy.

To detect differences between the final sample (*n* = 68) and respondents who were excluded (*n* = 25), comparative analyses on available demographics and key metrics were conducted. Continental distributions showed no significant differences (χ^2^(3) = 5.5794, *p* = 0.1340). The final sample was from Europe (*n* = 25, 36.8%), North America (*n* = 22, 32.4%), Oceania (*n* = 19, 27.9%), and Asia (*n* = 2, 2.9%). The non-completers were from Europe (*n* = 14, 58.3%), North America (*n* = 4, 36.8%), Oceania (*n* = 4, 36.8%), and Asia (*n* = 2, 36.8%). The gender distribution of the final sample was non-binary/non-conforming (*n* = 1, 1.5%), women (*n* = 38, 55.6%), and men (*n* = 29, 42.5%), while that of the non-completers was non-binary/non-conforming (*n* = 1, 4.0%), women (*n* = 17, 68.0%), and men (*n* = 7, 28.0%). Although there were proportionately more women than men among the non-completers, the difference between the distributions was not statistically significant (χ^2^(2) = 2.010, *p* = 0.3660). The final sample was on average slightly younger (M = 41.4 years, SD = 10.3 years, median = 40 years, range 25–65 years) than the non-completers (M = 47.4 years, SD = 9.4 years, median = 46.5 years, range 33–73 years). This difference was statistically significant (T(89) = 2.5019, *p* = 0.0142). However, the difference in work experience among the final sample (M = 10.3 years, SD = 9.0 years, median = 7 years, range 0–40 years) and the non-completers (M = 8.4 years, SD = 8.3 years, median = 6.5 years, range = 0–30 years) was not statistically significant (T(88) = 0.889, *p* = 0.376). In sum, these comparative analyses revealed no systematic differences between completers and non-completers, although completers were slightly younger than non-completers.

### 2.3. Data Analysis

#### 2.3.1. The Quality of the Survey Data

The elicited data varied in richness and length, altogether comprising over 11,000 words of descriptive and reflexive content. All responses were provided in understandable English, except for one response, which was submitted in French and translated to English by a university lecturer upon inclusion. The open-ended question had no word limit; however most responses were concise (i.e., shorter than 300 words), counting less than 100 words (*n* = 28), between 100 and 199 words (*n* = 20), and between 200 and 299 words (*n* = 12) respectively. Six responses were lengthier, counting between 300 and 699 words, whereas the two longest texts counted 826 and 1057 words precisely. Data were extracted and anonymized from Survey Monkey into Microsoft Word, where the cleaning and processing was carried out manually, preparing for the analysis.

#### 2.3.2. The Application of Constructivist Grounded Theory Methodology

The analysis was conducted according to a constructivist grounded theory approach as outlined by Charmaz [34]. The inductive phase consisted of an initial, open coding, which entailed labeling snippets of data to take them apart, while being attentive to its meaning and content. Moving into the deductive phase, the material was restructured into 16 tentative analytic categories. Focused codes were then identified based on how these initial codes would coalesce, each code accounting for more data to ensure representativeness, whilst still maintaining the richness of the material. Tentative focused codes were further pursued into delineating meaningful practice components, whilst retaining a strong foundation in the data. Theoretical coding entailed the elicitation of a proposed conceptual framework arriving at a progressive level of abstraction [36], which in this case meant to refine and finalize the eight practice components in a temporal structure and list key descriptive features of each component. Finally, the proposed practice theory was compared to the existing literature and assessed according to the four quality criteria: (a) credibility, (b) originality, (c) resonance, and (d) usefulness [34,37]. During all analytic phases, the first author (C.R.F.) wrote successive memos, which supported the abstraction of ideas [34] upon discussions with co-authors.

A constructivist approach to grounded theory analysis acknowledges interpretation in theory building and therefore brings in the subjectivity of participants and researchers alike, all situated in their social, historical, local, and interactional contexts [28]. The research team comprised one woman and three men of Norwegian, Finnish and Canadian descent. All authors have graduate level education, including PhDs in Health Science (C.R.F.), Psychology (M.M.), and Education (N.J.H.), and a specialist psychologist within Developmental Psychology (P.L.). All authors have been involved in outdoor therapies research and/or practice on national and international levels individually for between 14 and 30 years. This insider position provided the research team with necessary domain knowledge to interpret practitioners’ descriptions of implicit theories, while simultaneously requiring reflexivity to avoid imposing their own practice frameworks onto the data. Throughout the analytical process, constant comparative methods and team discussions were employed to challenge individual interpretations and maintain fidelity to participant voices. Still, interpretations and co-construction of knowledge are unavoidably influenced by the authors’ professional (health science, psychology, education, counseling) and cultural (Nordic and North American) backgrounds, as well as the shared commitment to advancing evidence-based and ethically grounded outdoor therapy practice, theory, and research.

#### 2.3.3. Ethics

Participation in the study was voluntary, and all survey respondents provided their consent. Participants were informed of the possibility to withdraw at any time without having to provide an explanation. Ethical approval was obtained from the University of Victoria Human Ethics Board (no. 22-0257). Contact information was provided to enable participants to forward any questions or concerns they might have. Participation in the study could be beneficial on an individual level in terms of fostering personal reflection concerning guiding frameworks in their practice, and on a societal level by contributing towards theory-generating research to inform and improve outdoor therapy practice, training, and policies, ultimately intended to benefit participants across outdoor therapies.

## 3. Results

**Figure 1:**
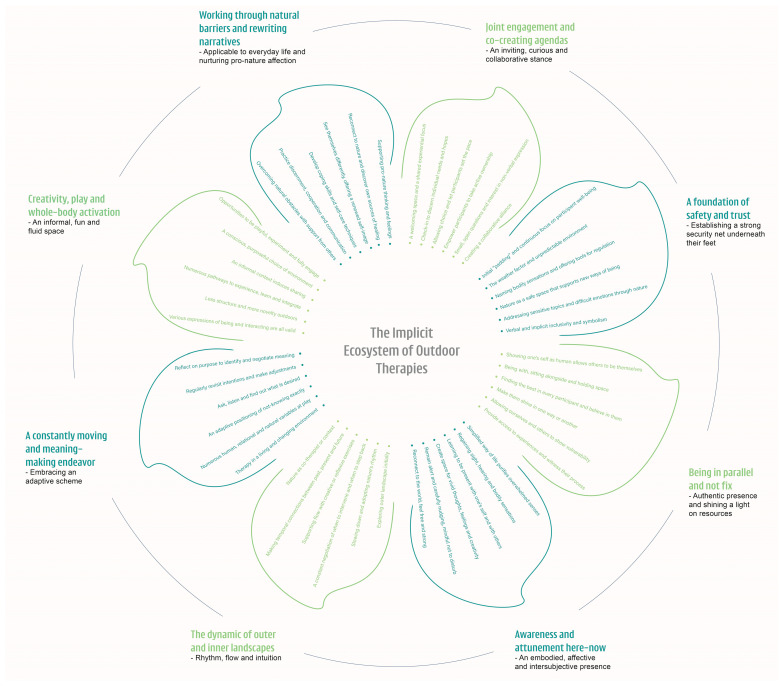
The implicit ecosystem of outdoor therapies.

Based on responses from 68 outdoor therapy practitioners representing 18 nations, a constructivist grounded theory analysis resulted in a proposed theoretical framework where eight practice components altogether form an interconnected circular system, comprising: (1) joint engagement and co-creating agendas; (2) a foundation of safety and trust; (3) being in parallel and not fix; (4) awareness and attunement here-now; (5) the dynamic of outer and inner landscapes; (6) a constantly moving and meaning-making endeavor; (7) creativity, play, and whole-body activation; and (8) working through natural barriers and rewriting narratives. Each practice component includes a descriptive subtitle and six key explanations, altogether representing a proposed practice theory, called *the implicit ecosystem of outdoor therapies* (see Figure 1 below).

As we present the findings, outdoor therapy practitioners are referred to as respondents, therapists, or facilitators, whereas participants in outdoor therapy practices are referred to as participants or clients. All excerpts are numbered to reflect the representativeness amongst respondents.

### 3.1. Joint Engagement and Co-Creating Agendas: An Inviting, Curious, and Collaborative Stance

The starting point of a therapeutic process, oftentimes entails establishing a shared experiential focus between facilitator and participants, here described as a meeting between two selves: “Firstly, I seek to achieve a joint engagement where we combine our interests and absorb ourselves in the task. This helps them experience my sense of self, which may help to establish their sense of self” (#67). Such a shared engagement appears to enable a connection between facilitator and participant. This initial connectedness should grow from a place of mutual respect, where conversations are signified by turn taking and active listening (#63) supported by an openness and curiosity, explained here by one facilitator: “I make sure my invitations to go out and connect to nature are in form of small open curious questions” (#4). Nature was considered a particularly suitable environment for this joint engagement, where one respondent stated that: “Outdoors is a natural classroom for curious people” (#49). Several respondents described outdoor therapies as an inclusive, collaborative practice, emphasizing the importance of starting each session with a check-in to discern individual needs and wishes:

For my groups, we do a lot of work around making a space where people feel they can come no matter how they feel that day […] We start our days with check-ins and then decide as a group what we can cope with, and how everyone can get what they need from the day.

(#17)

This co-creation of an agenda for many entailed establishing a: “Collaboration with clients on the recovery pathway and letting them set the pace, both physically and emotionally” (#35), as such empowering participants to take active ownership of their trajectories, as stated, “As often as possible, I work to allow the participant some choice in how they interact and participate” (#1). Calibrating the degree of participant influence and facilitator support was described as “hard to gauge exactly” (#25), although of great importance: “How much choice and control people have about their participation feels really important” (#25). Furthermore, through the application of a curious stance, therapists can gain valuable implicit knowledge about each individual participant: “When participants engage in an activity I become interested in what they do, say and express in non-verbal ways. How they respond to others and being curious about how what they are noticing about themselves occurs in other settings” (#1). Finally, shared outdoor activities were considered a conducive pathway for building a therapeutic alliance with participants, which often seemed to occur faster in nature than indoors: “Engaging with the client in experiences like a hike, climbing a tree or other shared experiences creates a deeper therapeutic connection in much more rapid ways than in a clinical setting” (#36). A collaborative alliance was described as an important starting point for the continued therapeutic process and to ensure the second component—a safe foundation for outdoor therapies.

### 3.2. A Foundation of Safety and Trust: Establishing a Strong Security Net Underneath Their Feet

A primary task in outdoor therapies, reported by respondents, was to establish a sense of safety and trust as a foundation for their practice. Safety was a priority from the start, explained as such, by one respondent: “At the beginning of every trip, ‘padding’ around our participants so they can feel there’s a strong security net underneath their feet” (#28). The aforementioned joint engagement was another venue to develop a trusting alliance: “Engaging with my clients outdoors facilitates side-by-side coaching. This also provides an opportunity for the clients to develop trust” (#52). However, nature is a semi-unpredictable environment and conditions can change rapidly, to where safety measures must be a constant concern:

The weather conditions can change the rhythm of the session completely and make it unsafe […] Sometimes they are able to name their feelings and bodily sensations and sometimes I need to step in with regards to their physical and emotional safety.

(#36)

Indeed, a foundation of safety and trust is not a static or single event, therefore a continuous focus on participant well-being is emphasized: “Maintaining a safe comfortable space for all throughout is essential” (#33). Several techniques and tools were mentioned that facilitators could use for calming and regulation when needed, for instance: “Having an opportunity to put up a hammock between trees to create safety from the ground or to regulate the nervous system can be crucial for some of my clients that tend to dissociate if things become too challenging” (#36). Despite demanding conditions outdoors from time to time, nature was depicted as a preferred setting for therapeutic work: “For some of my clients, nature seems to provide more safety than inside” (#36). By offering a sense of acceptance, nature can support new ways of being:

I feel that the living nature provides my clients with a sense of belonging as it welcomes all and accepts all kinds of emotions and slowly supports the peeling of unhelpful patterns through creating enough safety for them to try out new helpful patterns in the rewiring of the brain.

(#36)

Furthermore, sensitive topics can be indirectly addressed through nature, which for some would feel like a safer or more natural approach: “Connecting difficult emotions through metaphors and constellations with natural elements instead of using words can sometimes be the first safe ways of addressing difficult experiences” (#36). Sometimes safety can come through not using words, whereas other times deliberate and sensitive use of words is necessary in order to establish safety, as emphasized by a respondent working in a bi-cultural environment: “I now spend more time getting to know each other and use more inclusive language and symbolism” (#14). Both verbalized and implicit inclusivity can be essential parts of the security net, which is also relevant for the third component that entails authenticity and focusing on participant resources.

### 3.3. Being in Parallel and Not Fix: Authentic Presence and Shining a Light on Resources

Several respondents described their positioning as “being in parallel” (#19) and “being-with” (#19), reflecting a sense of equality between facilitators and participants, and emphasizing a relational presence. Authenticity was another important quality of the facilitator stance, which invited authenticity in return: “I tend to be natural, to show myself as human, just like I am. I think that provides comfort and allows clients to be themselves” (#10). Another dimension of the facilitator stance was illustrated by the metaphors of sitting alongside and not having to “fix” anyone, instead focusing on bringing out the resources of each participant:

I work on my way of being—authentic presence and mindfulness—facilitation and asking powerful questions, holding space […] I believe these are the most important skills that are required in this work. To be able to sit alongside and not to fix. To believe that the person has their own resources, and my job is to help uncover them, to help shine a light on them.

(#18)

A similar resource-focus was mentioned by several respondents as an identified objective of outdoor therapy practitioners: “Finding the best out of every participant, believe in them, and make them shine one way or another” (#28). Nature environments were similarly suggested to bring out the best in facilitators: “Being in nature allows me to offer the ‘best version’ of myself as a therapist because I feel good being in nature” (#34). An authentic presence, however, did not merely entail preferred versions of oneself, it also meant: “Allowing ourselves and others to show vulnerability” (#28). The facilitator positioning could be demanding at times, while at other times it felt almost effortless; “being in parallel” meant maintaining an attentive presence and offering support along the way:

At times, it feels so effortless, and my role seems almost superfluous. As if they would have reached that realization themselves, if in nature. Most often, I find I am able to provide access to an experience that they may not have felt comfortable doing themselves. My skills as an outdoor adventure leader bring them into nature; and my skills as a practitioner hold space for them to find themselves, wherever they happen to be in that moment. And in the end, when we part ways, they think they accessed that knowledge, or sense of calm, or awareness, themselves, in nature, I was just a witness to their process.

(#15)

This “being-with” is not a passive bystander stance, rather, it demands an engaged attentiveness and presence in order to “really be with our participants” (#28), which is described in the next component.

### 3.4. Awareness and Attunement Here-Now: An Embodied, Affective, and Intersubjective Presence

In nature, everyday distractions are often removed, which can enable both practitioners and participants to be present and to recover:

I love to be outdoors. I can be the most in the ‘here and now’. I believe the same thing happens to my clients as well […] I believe this simplified way of life purifies the technologically overwhelmed senses of human beings, as they would regain their sight, their hearing and bodily sensations.

(#11)

The outdoors was suggested to provide numerous opportunities for, and variations of, attunement, described as: “Embodied and affective connection, conscious or less conscious, between the person and the environment” (#44). Implicit awareness can occur on many levels and amidst the triangular exchanges between the nature environment(s), participant(s), and facilitator(s), where the facilitator stance entails to: “Try to be alert when the client realizes something, and/or understands. Perhaps not cognitively, but intuitively” (#54). Both facilitator and participant presence are deemed important implicit domains in outdoor therapies, which allow for multiple connections:

Working outdoors, I especially believe presence is one of the most powerful effects. Nature allows the client to stay focused here-and-now, which influences their way of thinking and acting. It opens up to sensory experiences, where the client is able to connect with themselves in a different manner than what they are used to. This also allows connection with emotions, body, thoughts and others around. The client is not only learning to be present with themselves, but also with others.

(#41)

Most of these processes are likely to be implicit in nature and part of the tacit repertoire of outdoor practitioners, which may offer rather unique opportunities for relational and sensory therapeutic work, according to respondents:

For me, nature is an indivisible entity […] in which relationships between people can be shaped and reshaped and in which relationships can be worked on. Part of that whole is essentially the human–nature relationship. Attunement to nature’s rhythms, intensities and to other sensory perceptions creates space for vivid thoughts, feelings and creativity that bring in opportunities to work on difficulties.

(#44)

The facilitator’s role may be to carefully nudge these processes, whilst all along being mindful not to disturb or interfere, guided by their attunement and implicit relational knowing:

My task as a therapist is to make this setting possible and with minimal facilitation to guide the clients into their important themes. I tend to keep my voice down as much as possible, my utterances quite restricted, feedback also quite neutral. On tracks, I show the way but set myself walking behind the clients to give them the freedom of first impressions. It is of utmost importance to not disturb the relational processes, impressions and experiences of the clients with questioning and commenting in wrong moments, to not ‘steal’ or diminish their experiences […] In cases of strong experiences of awe or other touching events, I leave momentary reflection out, since reflecting especially verbally moves a person away from the emotional experience. Or from sensation to analytical intellectualization and then the ‘now-moment’ is gone. What nature also offers or supports is the feeling of here-now, that for some people seems to be groundbreaking in their process of change.

(#44)

These moments of attunement in nature may provide a therapeutic environment where participants: “Tap into different parts of themselves and allow them to build a relationship with their senses and thinking” (#37), which is furthermore proposed to “Help the clients to reconnect to the world, to sense the world around them, feel free and strong” (#39). This interaction of nature—the outer landscapes—and human nature—referred to as the inner landscapes—is another implicit dynamic of outdoor therapies, which makes up the fifth component of the ecosystem.

### 3.5. The Dynamic of Outer and Inner Landscapes: Rhythm, Flow, and Intuition

Outdoor therapies are at times offered when participants are not comfortable with, or not improving in, indoor or talk-based provisions of therapy. When recontextualizing therapy to a nature setting, the focus can be purposefully directed outwards at first: “With some clients who are less open to counseling or talking directly about their inner world, walking along side, exploring something interesting in the outer landscape before inviting exploration of their inner world” (#65). Depending on individual needs and the aforementioned co-created agenda, the initial phase can entail a slowing down at first before embarking on a paced route towards exploring inner landscapes: “Bringing participants to slow down, to adopt nature’s rhythm, to focus on their senses, to meet with their emotions, to guide this awareness, to disconnect from technology, to let go” (#28). As part of the facilitator stance, a certain implicit dynamic is constantly negotiated: “So, there is a sort of flow, of knowing when to push and be more directive, or to sit with and patiently hold the space for the client, such as therapeutic silence” (#63). This assessment of when to intervene and when to hold back seems to be one of these implicit movements between outer and inner landscapes, guided by facilitator intuition:

There maybe is something there about using intuition to guide when I am the facilitator and when nature is speaking. When is it best for me to ask a question and say something and when is it best to let nature speak and not interrupt. I guess in talk therapy there is the question of when to say something and when to be quiet—which exists outside too, but there are more questions […] when to be active, when to be still, when to push, when to remain passive […] and on and on the option goes. So there is a lot of intuition that guides these choices, I guess.

(#25)

For some facilitators, flow seems to refer to this movement between an outer and inner focus, where various activities can be applied to support this dynamic, such as creative exercises, as stated, “I use art in nature to deepen self-analysis and to help them into a flow state” (#33), or facilitated alone time, which is suggested to enable temporal connections:

Using solo moments can often create a certain flow in the client’s reflective activities whether it is through reflective writing or doing. In this flow, they often experience these a-ha moments and make connections from the past, to the present and the future.

(#36)

Several respondents emphasized the importance of flow states, with some suggesting that it was precisely in these moments that change would occur. The timeframe and format of outdoor therapies are other dimensions that play into this dynamic, because it can take time for a participant to become immersed. Also, time restrictions of single sessions can sometimes mean that the facilitator needs to interrupt or close flow processes, instead of allowing a natural conclusion. Finally, it is important to point out that the views with regards to the role of the outer landscapes—or nature—is diverse amongst outdoor therapy practitioners. Whereas some respondents consider nature to be an active co-facilitator in their practice, others express a more passive view of nature as merely a landscape:

While some have argued that nature is the co-therapist, I’m not sure that I agree […] I find the concept of nature having a role to be similar to indoor therapists arguing about the role of the coach or how to decorate a counselling room. The outdoors is simply a different place to do the work, and the active bodily engagement is a way I find useful for conducting therapy.

(#64)

Despite facilitators’ subjective perception of nature, the outdoors is arguably different from most therapeutic settings, as nature is a living entity unlike a piece of furniture, and thus always somehow in movement, which brings us to the sixth component.

### 3.6. A Constantly Moving and Meaning-Making Endeavor: Embracing an Adaptive Scheme

Outdoor therapies take place in environments that are constantly changing. From micro-level activity not visible to the human eye, to changes in weather, seasons, cycles, and time of day, involving countless living plants and creatures, nature environments afford a very different context for therapeutic work compared to most indoor settings: “In nature something always happens, something interesting, involving, motivating” (#11). Group interventions represent additional relational constellations constantly in movement, where facilitators and participants alike are subjected to a semi-unpredictable reality in outdoor therapies, which entails: “Not knowing […] often times not knowing exactly” (#2). In this constantly moving endeavor, numerous human, relational, and more-than-human variables are at play:

In my practice, I always aim to keep the participants in an adaptive scheme, in search of identifying patterns and strengths not yet known or that can be reinforced to help participants in their recovery. I will play with the various variables available to achieve this.

(#5)

This adaptive positioning and improvisational mode must, to a certain extent, be ingrained in outdoor therapy practitioners. Amidst this adaptive scheme, one respondent emphasized the importance of repeatedly checking in with the participants: “Ask, listen and find out what is desired. Some people want to simply sit and listen to the environment around them, some want something new, some want a lot of reflection, some want a lot of activity and movement” (#53). Without asking and paying close attention to the participants there could be a mismatch between content and what is needed, which could be prevented by regularly revisiting the initial intentions: “Sometimes stopping to think of the reasons we were doing this in the first place and how this activity related to it” (#2). Adjusting the tempo and distance during more strenuous physical movement can, for instance, be another practical tool to adapt to participants’ capabilities and revisit intentions:

When walking in the mountains with my clients I adjust the pace and the length of the walk to them and this influences my clients. If we slow down many of the important conversations happen. Also, while we rest there is a time to reflect on what is important for them […] and what is difficult.

(#56)

It seems that in these reflective moments, connections are made between content and purpose, as a negotiation of meaning, which is considered an essential part of the therapeutic process: “Meaning-making of the experiences we carry out is vital as part of the intentionality of our use of outdoor health care” (#61). Further, different ways of being together may hold different meanings, where for some, the more informal and playful aspects of outdoor therapies are particularly important, which make up the seventh component of the proposed ecosystem.

### 3.7. Creativity, Play, and Whole-Body Activation: An Informal, Fun, and Fluid Space

Several respondents mention the importance of creativity, individuality, and fluidity, as such allowing for various expressions of being and interacting in nature: “It’s a setting that welcomes different ways of participating, all valid. I feel there is space for more creativity, there’s the chance to ‘find your own space’ and there’s less structure and more novelty, the cohesion is more fluid outside” (#8). As such, outdoor therapies can offer numerous pathways to integrate and learn through experiential interactions:

Humans are wired to learn through play. A solely language-based interaction is flat, compared to the impact on behavior and memory of a shared experience […] Integrating physiological states and feedback from the body with our mental experience while engaged in experiences is more sensible to me than a more static shared experience.

(#19)

Moments of fun appeared to hold an inherent importance for some respondents, stating that: “There’s always humor and jokes” (#10) and “Giving space to moments of pure fun, with no other intention than laughing hard” (#28). The degree of therapeutic depth appeared to vary across responses, where some intentionally remained on a surface level: “I consider my work to be something like ‘hangout with purpose’. I like to have fun and stay on surface with clients” (#64). However, a play-based approach can still initiate embodied and intersubjective realizations: “Play between sunshine and shade for both somatic noticing and insights between being seen or ability to see” (#3). Furthermore, the openness of nature was suggested to offer a less restrictive space, where unstructured moments in outdoor therapies could: “Put in place an informal context where clients share in a generous way” (#29). Depending on an intentional choice of nature setting, numerous therapeutic opportunities can be accessed through whole-body activation:

A conscious, purposeful choice of environment is, in my opinion, very important. I consciously change the physical environment in order to create new, unique experiences for the participants themselves and give them the opportunity to look at similar things from a different perspective. For example, a conversation while walking along the sea and a conversation in the office will be very different. The beach naturally offers an opportunity to balance, providing different, new sensory impulses, an opportunity to be playful, to experiment, to spread your focus, to look into the distance. In such an environment, the whole body is engaged much more fully, which I think is very important.

(#13)

At last, arriving at the final component of the proposed implicit ecosystem; working through natural obstacles that are applicable to participants’ everyday lives, may yield experiences that can alter their life stories.

### 3.8. Working Through Natural Barriers and Rewriting Narratives: Applicable to Everyday Life and Nurturing Pro-Nature Affection

In outdoor therapies, participants experience natural barriers that occur in real-life situations, whilst ideally having in situ support readily available from facilitators and co-participants:

When working in nature, various natural obstacles regularly arise, which need the support of others to overcome. In my opinion, in a clinical environment, such barriers must be specifically created and can seem artificial, so I much prefer natural barriers that allow you to work with expressing your needs, asking for and providing support, cooperation and communication skills. Such obstacles usually also serve well as metaphors to adapt to everyday life.

(#13)

Through overcoming obstacles and acquiring new experiences, participants may develop coping skills and self-care techniques they can make use of in their everyday lives: “Moving the therapy room outdoors lets the client collect tools to bring into their own every day […] they are able to discover therapeutic potential that they can also apply in their own lives” (#41). Further, working through barriers may foster a renewed self-image: “A time where the client can see themselves differently” (#64). To increase transference, a facilitator task can entail to: “Consolidate those feelings and inspire them to notice these differences in their lives outside of therapy” (#64) and furthermore supporting them in what may be understood as a: “Process of becoming, to overcome fear and facilitate trust, to assist them in rewriting the narrative or stories they tell themselves” (#63). Final outputs from outdoor therapies may include not only renewed self-appreciation, but also a deep appreciation for nature and a sense of belonging, where some facilitators draw on ancestral traditions:

My role is to reconnect people to the natural world so that they can discover their own sources of healing, by tuning into ancestral wisdom. We build relationships with the plants, creatures, rocks, water, wind […] We learn we are never alone.

(#22)

This deep connectedness to nature is suggested to nurture “pro-nature thinking and feelings in people” (#44), which is proposed to be an inherent agenda for outdoor therapy practitioners: “Our life as all life depends on the well-being of nature, so I want to create situations that might raise people’s affections toward nature. I deeply feel this is my responsibility” (#44). On that note, the eight practice components of the proposed ecosystem of outdoor therapies are presented and shall now be considered according to the existing literature and quality criteria.

## 4. Discussion

The proposed practice theory, the implicit ecosystem of outdoor therapies, is grounded in elicited data collected from 68 self-identified outdoor therapy practitioners representing 18 nations across four continents. This implicit ecosystem is intended to conceptualize a synergistic whole and as such offers a holistic framework for outdoor therapy practice, theory, research, and training. The practice theory comprises eight interrelated components, each making up a leaf of the conceptual flower with six descriptive key properties. In line with a constructivist grounded theory approach, the final stages include a consideration of how the proposed theory resonates with the existing literature, followed by a quality appraisal according to four criteria. The purpose of integrating the practice theory with existing psychology literature is to identify potential avenues for further theoretical and scientific exploration. The objective is not to validate the proposed theory empirically, but rather to explore conceptual alignments and knowledge gaps that may inform future hypothesis-testing research. The following discussion examines six psychological constructs: (a) self-determination, (b) equality, (c) connectedness, (d) dialogicity, (e) self-efficacy, and (f) sustainability, which is suggested to resonate with the eight practice components of the practice theory. Finally, the four quality criteria of a constructivist grounded theory are addressed, which delineates the implications and limitations of this study.

The first construct, self-determination, is a familiar concept within psychology and motivation literature, which rests on a well-established explicit framework, called self-determination theory (e.g., [38]). Self-determination refers to each person’s ability and power to make decisions for themselves and thereby exert influence on their own situation and recovery trajectory. There are at least three reasons why this construct resonates with the proposed therapeutic ecosystem and could offer interesting avenues for further investigations. First, the importance of participant choice and control was prevalent across the dataset. Yet the implicitness that goes into ensuring that self-determination is upheld and not jeopardized across activities and changing conditions in outdoor health care is significant; therefore, this aspect of outdoor therapy practice should be further investigated and explicated through best practice guidelines. Indeed, the autonomy of each participant should be ensured across all outdoor therapies; therefore, the two first practice components of the proposed conceptual framework, entailing the co-creation of agendas in a joint collaborative stance between facilitators and participants, that is furthermore securely based on a foundation of safety and trust throughout, are essential for each client’s self-determination.

Further, self-determination relates to the second construct, equality, which refers to the rights of all people to be treated fairly and have the same opportunities. Arguably, a large degree of implicitness in terms of power dynamics and potential inequalities exist across therapeutic practices in general, and outdoor therapies specifically, to the point where these dimensions should be closely assessed across practice, training, and research. In the context of this study, equality also refers to the ideal reciprocity and lack of hierarchy in the triad between participants, facilitators, and nature. Whereas an anthropocentric positioning places humans over nature, outdoor therapies seek to not only foster a between-human connection, but also an affection towards the more-than-human nature, often referred to as nature connectedness [39]. This renewed appreciation for and deep connectedness to nature is emphasized in the final component of the ecosystem, suggesting that nurturing of pro-nature affection is an inherent task for outdoor therapy practitioners. These considerations overlap with the proposed third construct, connectedness, which refers to both within- and between-human contact and affiliations with other living beings and nature environments [40,41]. However, this construct carries considerable implicitness in terms of the myriad ways it could be comprehended and implemented by outdoor therapy practitioners and let alone experienced by each individual participant. As such, it is a complex concept, representing various forms and degrees of attachment to oneself, to co-participants, and facilitators, as well as to our natural habitat, within the outdoor therapy ecosystem. The next proposed construct can be applied to navigate this relational and environmental web.

The fourth construct, dialogicity, represents the communicative and relational properties of connectedness and reciprocity through an emphasis on our embodied being in the present moment [42]. The core of dialogicity entails unconditional respect for all living beings and ensuring a space for all ‘voices’ to be heard [43]. Clinicians are often trained to carry out interventions according to preset plans and treatment goals informed by assessments and clinical procedures. However, rigid approaches could come in the way of an authentic presence—the “being with” each other—including a deep sense of listening and implicit relational knowing. While a dialogical approach has been developed as a therapeutic method, it is also introduced as a way of life, where intersubjectivity is suggested to form the basis of our human experience [42]. Dialogicity offers a useful construct to navigate the (eco)systemic components referring to the dynamic of inner and outer landscapes, and the adaptive scheme of constant moving and meaning-making.

Fifth, self-efficacy may feature as both a psychological construct and a treatment outcome within outdoor therapies, drawing from positive psychology (e.g., [44]) and self-efficacy theory (e.g., [45]). Self-efficacy refers to a person’s belief in their own capabilities, which is closely linked to a sense of agency and overall daily life functioning. Overcoming natural, relevant barriers in an outdoor therapy setting can be a vehicle for strengthening this belief, developing competence, and nurturing a capable self-image. The degree of transference and sustainability of these acquired beliefs and competencies into a rewriting of narratives are avenues for future investigations within the field of outdoor therapies. Also, there are numerous dimensions of self-efficacy across cognitive, affective, embodied, and relational dimensions that can be investigated along multiple pathways within outdoor therapy practices. Most of these mechanisms are largely implicit in nature and yet to be subjected to scientific scrutiny [32].

The sixth proposed construct, sustainability, may hold several connotations within the field of outdoor therapies, referring firstly to a psychosocial dimension, in terms of the ability to sustain positive development over time. In many instances, outdoor therapy practices integrate home environments and local communities, and as such involve family members, school-, and/or work-related affiliations to strengthen the likelihood of transference and sustainability over time and across daily life settings. Secondly, in terms of safeguarding sustainability for outdoor therapy providers, this could entail always striving to ensure high quality provision (see [4], for sustainability indicators when utilizing nature for mental health). Finally, and perhaps most commonly, sustainability refers to maximizing environmental awareness and minimizing harm to the environment, which remains to be made even more explicit across outdoor therapy practices internationally as a crucial step to advance this field of practice, where the findings of this study emphasize the responsibility of outdoor therapy practitioners to nurture pro-nature affection and action.

Within a constructivist grounded theory methodology, Charmaz [34,37] identifies four quality criteria, including: (a) credibility, (b) originality, (c) resonance, and (d) usefulness. Credibility relates to the size and quality of the dataset, whether the collected data suffices to answer incisive questions and develop a thorough analysis [35]. Another dimension of credibility is the researchers’ abilities to maintain a reflexive and critical stance throughout the analytic process. When it comes to the scope and relevance of the elicited data in this study, both richness and depth of the material were more than satisfactory for the purpose of conducting an initial mapping of the implicit terrain of outdoor therapies and the subsequent construction of a preliminary grounded theory. The results section integrates numerous respondent excerpts to make the analysis and findings transparent and credible. The composition of the research team, made up of four experienced practitioner-researchers representing different backgrounds in terms of age, gender, nationality, educational background, and experience, was intended to increase reflexivity and ensure domain knowledge. At the same time, for three members of the research team and many of the respondents, English was not their native language, which can be a limitation because expressing implicit content in a second language could compromise the provision of detail and cultural accuracy. However, for the purpose of constructing a practice theory of international relevance, conducting the mapping in a shared language was deemed the most feasible and relevant option.

To the best of our knowledge, this is the first study that applies a grounded theory methodology and an ecosystemic lens to explore and describe the tacit landscape of outdoor therapies. This bottom-up approach of building theory from practitioners’ descriptions and providing an original conceptual framework is a unique contribution within the field of outdoor therapies that is applicable to practice, training, and research. Focused attention towards noticing and attempting to explicate practice components not yet fully appreciated, emphasized, or even articulated is hoped to be of great value, and hence useful, to the collective knowledge base for theoreticians, researchers, practitioners, and educators, that is intended to ultimately benefit participants in outdoor therapies. Finally, implicit theories can be right and helpful, as well as wrong and harmful; thus, experiences related to both scenarios should lead to further modifications, contributing to collectively increasing the accuracy, resonance, and usefulness of the proposed practice theory over time and across cultural contexts.

## 5. Limitations of the Present Study

Several limitations of this study warrant consideration. First, the sample was recruited through snowball sampling via professional and social media networks, which, while appropriate for an emergent and heterogeneous field, introduces a self-selection bias. Participation was voluntary and dependent on individual availability, willingness, and online access, meaning the sample cannot be considered fully representative of the global outdoor therapy workforce. The absence of a comprehensive global register of outdoor therapy practitioners makes it impossible to verify proportional representativeness across countries or professional approaches.

Second, the geographical distribution of respondents is skewed toward English-speaking nations with well-established professional networks, most notably Canada and New Zealand/Aotearoa. Although the study encompasses practitioners from 18 nations across four continents, practitioners from regions with less-developed professional infrastructure are likely underrepresented, which may limit the cultural generalizability of the proposed practice theory.

Third, data collection relied on a single written open-ended question. While this yielded rich diversity of responses ranging from 40 to 1057 words, adding questions or collecting data through individual interviews might have produced different results. Using the written form may be especially constraining for capturing tacit and embodied knowledge, which by its nature resists verbal articulation.

Fourth, the framework is derived exclusively from practitioners’ self-reported accounts and therefore reflects professional perception rather than empirically measured therapeutic effectiveness. The practice theory makes no claims regarding causal relationships between the proposed components and therapeutic outcomes, and should not be interpreted as doing so. Further research employing diverse methodologies, including observational studies, client-reported outcomes, and mixed-methods designs, is needed to examine the mechanisms and effectiveness of the identified components.

Fifth, while the analysis followed a rigorous constructivist grounded theory approach and the final eight-component structure reflects theoretical sufficiency for this dataset, it is not claimed to represent an exhaustive structural mapping of all possible dimensions of outdoor therapies. Theoretical saturation is difficult to establish conclusively in an emergent field, and the framework should be understood as a preliminary practice map, open to modification as new evidence, contexts, and voices from more diverse practitioner populations contribute to its refinement and further adaptations.

Finally, the positionality of the research team—four experienced practitioner-researchers from Norwegian, Finnish, and Canadian contexts, all with a shared commitment to advancing outdoor therapy—constitutes both a strength in terms of domain knowledge and a limitation in terms of the risk of imposing existing practice frameworks onto the analysis. Although constant comparative methods and team-based reflexivity were employed throughout the analytical process, interpretations remain inevitably shaped by the researchers’ professional, cultural, and linguistic backgrounds.

Despite these limitations, the present contribution is a first step toward articulating that which is difficult to articulate and to explicate that which is challenging to put into words. Outdoor therapies are an embodied and intuitive endeavor through-and-through, and further research based on participant observation, physiological measurements, and qualitative interviews are likely to shed further light on this important, but until now underexplored theme.

## 6. Conclusions

The purpose of this international survey-based study was to generate theory grounded in descriptions of practice frameworks from outdoor therapy practitioners, with a particular focus on exploring the implicit and less articulated aspects of this diverse discipline. Findings were presented across eight integrated practice components of the proposed practice theory, the implicit ecosystem of outdoor therapies. Drawing a parallel to the study of complex adaptations and alterations of ecosystems over time within the field of ecology, conceptual frameworks such as the present practice theory should undergo context-sensitive and iterative revisions shaped by new settings and evidence. In line with the ecosystem metaphor, such adaptations should not be interpreted as a linear succession from ‘early’ to ‘mature’ stages, nor as implying that later versions are inherently better.

Presently, this theoretical framework offers a practice map that is constructed from practitioners’ subjective accounts that describe the facilitator stance and the implicit web of relational and environmental components that guide the provision of outdoor therapies; as such the practice theory makes no claims to effectiveness nor causality. Future avenues for theoretical and scientific exploration include examining how the therapeutic components may interact to produce various outcomes across populations and contexts, and formulating testable hypotheses about potential causal interactions. A continued exploration of the tacit landscapes of outdoor therapies can further inform, adapt, and revise the proposed practice theory, contributing towards advancing theory, training, and research to support safe, intentional, and ethically mindful outdoor therapy practices worldwide.

## Data Availability

The datasets analyzed during the current study are available from the corresponding author on reasonable request.
